# Cardiac Effects of Thyrotropin Oversuppression with Levothyroxine in Young Women with Differentiated Thyroid Cancer

**DOI:** 10.1155/2016/9846790

**Published:** 2016-06-23

**Authors:** Kyung-Soon Hong, Jung-Woo Son, Ohk Hyun Ryu, Moon-Gi Choi, Ji Yeon Hong, Seong Jin Lee

**Affiliations:** ^1^Division of Cardiology, Department of Internal Medicine, College of Medicine, Hallym University, Chuncheon 24253, Republic of Korea; ^2^Division of Endocrinology and Metabolism, Department of Internal Medicine, College of Medicine, Hallym University, Chuncheon 24253, Republic of Korea; ^3^Division of Cardiology, Department of Internal Medicine, Hanil General Hospital, Seoul 01450, Republic of Korea; ^4^Division of Endocrinology and Metabolism, Department of Internal Medicine, Hallym University Sacred Heart Hospital, Anyang-si 14068, Republic of Korea

## Abstract

*Background*. We investigated the cardiac effects of TSH (thyroid-stimulating hormone) oversuppression in women with thyroidectomized differentiated thyroid cancer (DTC) during levothyroxine suppression therapy.* Methods*. Fourteen young female patients with DTC were enrolled. The duration of TSH-suppressive therapy was 5 to 9 years. They satisfied the following criteria: (1) a serum level of TSH < 0.1 mU/L in the intermediate-risk or TSH < 0.3 mU/L in the low-recurrence-risk group and (2) having been receiving a fixed dose of LT4 before the study. Controls matched for age, sex, and body mass index (BMI) were compared in terms of the levels of serum free T4, free T3, TSH, plasma N-terminal pro-brain natriuretic peptide (NT-pro-BNP), and cardiac functions and structures.* Results*. DTC patients and control subjects were well matched in heart rate and blood pressure. There were marked differences in serum TSH (*P* = 0.001) and free T4 (*P* = 0.002). However, there were no differences between the groups in serum free T3 and plasma NT-pro-BNP. Furthermore, there were nonsignificant differences in cardiac functions and structures between the groups.* Conclusions*. This study shows that TSH suppression therapy in women with DTC may be safe with respect to cardiac functions and structures despite intermittent oversuppression of TSH during long-term suppressive therapy.* Trial Registration*. This trial is registered with clinicaltrials.gov identifier NCT02645786.

## 1. Introduction

The global incidence of thyroid cancer has been rising steadily over the recent decade [1, 2]. In Korea, thyroid cancer was the most common type of cancer in the 2010 cancer registry (http://www.cancer.go.kr/upload/board/31817/179615_2.pdf accessed January 15, 2015). After total or near-total thyroidectomy in differentiated thyroid cancer, long-term thyrotropin (thyroid-stimulating hormone, TSH) suppression therapy with levothyroxine (LT4) should be carried out to prevent hypothyroidism and to minimize potential TSH stimulation of tumor growth in patients [3–5]. During long-term TSH suppression therapy, exogenous mild thyrotoxicosis or subclinical hyperthyroidism might develop and cause adverse effects on heart and bone metabolism [6, 7]. Several studies have reported that long-term TSH-suppressive therapy after thyroidectomy in cancer or goiter might induce increased resting heart rate, diastolic dysfunction, and increased left ventricular mass [8–10]. However, one study has reported that the cardiac function and structure of athyreotic thyroid cancer patients without thyrotoxic symptoms were not adversely affected despite receiving long-term TSH suppression therapy [11].

The American Joint Committee on Cancer (AJCC)/Union for International Cancer Control (UICC) TNM (classification of malignant tumors) system has been commonly used to predict survival in differentiated thyroid cancers (DTC) [12]. However, the prognosis of DTC is excellent. Therefore, it is more important to consider the risk of recurrence and adverse effects in long-term thyroid cancer management. The American Thyroid Association (ATA) developed a consensus-based clinicopathologic system for the assessment of recurrence risk. This risk stratification system is useful when guiding TSH-suppressive therapy. The ATA recommends that serum TSH should be suppressed for 5 to 10 years to low concentration levels (0.1–0.5 mU/L) in high-risk but clinically and biochemically disease-free groups or to the low normal range (0.3–2.0 mU/L) in patients free of disease, especially those at low risk of recurrence, in order to best balance benefits and risks [13–15]. The majority of papillary thyroid cancers tend to present between the ages of 30 and 50 years (http://seer.cancer.gov/statfacts/html/thyro.html accessed January 15, 2015). Given the same extent of disease, patients aged <45 years have a distinctly better prognosis than those aged ≥45 [16]. In those aged less than 45, the adverse effects of long-term LT4 therapy might occur less frequently than in older age groups because they have fewer comorbid conditions. Most DTC patients also fall into the categories of lower intermediate risk of recurrence. Therefore, when deciding the target of TSH suppression for individual DTC patients, the clinician needs to take into account any underlying comorbidities and the patient's age as well as the risk of recurrence.

When implementing chronic TSH suppression therapy with LT4, patients frequently encounter oversuppression relative to the target levels of TSH that correspond to their risk of mortality and recurrence. Therefore, the aim of this study was to evaluate the cardiac effects of TSH oversuppression in women with DTC during TSH-suppressive therapy for 5 to 9 years relative to their risk of recurrence.

## 2. Methods

### 2.1. Patients

From chart review, we selected 96 female differentiated thyroid cancer patients who received total or near-total thyroidectomy and thereafter regularly visited the Endocrine Out-Patient Department (OPD) of Chuncheon Sacred Heart Hospital. The majority of papillary thyroid cancers tend to present between 30 and 50 years of age (http://seer.cancer.gov/statfacts/html/thyro.html accessed January 15, 2015) and their risk of recurrence is low or intermediate. Menopause also might affect the cardiovascular risk factors in women. According to guidelines, the dose of thyroxine would be reduced after 10 years of thyroidectomy in DTC patients [13–15]. Therefore, additional enrollment criteria were as follows: (1) age less than 45 years when receiving total or near-total thyroidectomy, (2) serum level of TSH < 0.1 mU/L in the intermediate-risk or TSH < 0.3 mU/L in the low-recurrence-risk group [13, 14] over 2 years before study entry, (3) receiving TSH-suppressive therapy for 5 to 9 years with fixed dose of LT4 more than 2 years before study entry, and (4) no history of structural heart disease, arrhythmia, or cardiac symptoms (palpitation, exertional dyspnea, and chest discomfort) during therapy. Of the 17 patients who met the criteria, three patients did not consent to this study. Candidates who satisfied all the enrollment criteria took an electrocardiogram to rule out patients with arrhythmia. Finally, 14 DTC patients were enrolled and studied from September 2009 to March 2010. As each patient was enrolled, control subjects were selected from patients who visited the Endocrinology Department for thyroid nodule work-up. The control group had to meet the following criteria: (1) the subject matched to a patient by age (±2 years), sex, and body mass index (BMI) (±2 kg/m^2^), (2) levels within the reference range of serum TSH (0.3–4.6 mU/L), (3) no history of structural heart disease, arrhythmia, or cardiac symptoms, and (4) no history of comorbid diseases which affect thyroxine metabolism and cardiac structure, including hepatic or renal disease, anemia, and hypertension. All subjects who met the enrollment criteria took an electrocardiogram to rule out arrhythmias. Control subjects were recruited and tested from January 2010 to July 2011. The study protocol was approved by the Ethics Committee in the Chuncheon Sacred Heart Hospital (2009-19) before the study. All subjects provided written informed consent.

### 2.2. Assays

On the examination day, all participants were prohibited from smoking and consuming caffeine. After a light breakfast and medication, including LT4 in cancer patients, participants visited the hospital before 9 AM. We investigated the comorbid conditions of the participants. Body weight and height were measured while the subjects wore light clothing without shoes. The body mass index (BMI) was calculated as the weight in kilograms divided by the height in meters squared. Their blood pressure was taken after a 10-minute rest period. Subsequently, each subject underwent a 2-dimensional echocardiogram carried out by one examiner. After the cardiac work-up, blood samples were drawn for test of thyroid function. Blood samples were collected in prechilled tubes containing EDTA, immediately placed on ice, and promptly centrifuged at 4°C. After separation, plasma was stored at −80°C for the N-terminal pro-brain natriuretic peptide (NT-pro-BNP).

Serum thyroid function tests were performed by a chemiluminescent immunoassay (UNICELL DXI800, Beckman Coulter, USA). Serum TSH (reference value: 0.3–4.6 mU/L, detection limit: 0.0025 mU/L), free T4 (reference value: 7.0–20.0 pmol/L), and free T3 (reference value: 4.0–5.9 pmol/L) were measured. Plasma NT-pro-BNP measurements were done using chemiluminescent immunoassay method (Roche E170, Roche Diagnostics, Germany).

### 2.3. Echocardiography

Comprehensive transthoracic echocardiography was performed using commercially available equipment (IE33, Philips Medical System, Andover, Massachusetts). Standard 2-dimensional measurements were obtained as recommended by the American Society of Echocardiography (ASE) in the left lateral position [17]. Left atrial volume index was measured by the biplane area-length method. Left ventricular (LV) mass was calculated using the following equation: LV mass = 0.8(1.04{[LVIDd + PWTd + IVSd]^3^ − [LVIDd]^3^}) + 0.6, where LVID is LV end-diastolic dimension, PWT is posterior wall thickness, IVS is interventricular septal wall thickness, and d is diastole. Systolic function was evaluated by measurements of left ventricular ejection fraction (LVEF) and global longitudinal strain (GLS) of the LV. The following parameters of diastolic function were measured by tissue Doppler-derived imaging: early diastolic flow of mitral valve velocity (*E*), late diastolic flow of mitral valve velocity (*A*), early diastolic mitral annular velocity (*e*′), late diastolic mitral annular velocity (*a*′), and isovolumetric relaxation time (IVRT) were measured from the septal corner of the mitral annulus in the apical 4-chamber view. To evaluate the GLS of the left ventricle, echocardiographic images were obtained at the apical 4-chamber view and the strain was analyzed based on routine DICOM (Digital Imaging and Communications in Medicine) data sets using software (2D Cardiac Performance Analysis, TomTec, Munich, Germany). A region of interest was manually placed on the endocardial and epicardial borders. The echocardiographic data were gathered and analyzed by 2 experienced echocardiographers who were unaware of any corresponding clinical data.

### 2.4. Statistical Analysis

Data were reported as means and standard deviation (SD). For the comparison of means between groups, we used the Mann-Whitney test because the sample size was small (*n* = 14 for each group). All *P* values calculated are two-tailed and considered significant at *P* < 0.05. All statistical analysis was performed using SPSS 20.0 software (SPSS Inc., Chicago, USA).

## 3. Results

All cancer patients were of stage 1 in the AJCC staging system. 10 patients were classified as having intermediate risk and 4 patients fell into the low-risk-of-recurrence category. The mean LT4 dose was 2.77 *μ*g/kg (ranging from 1.67 to 3.82 *μ*g/kg). The baseline characteristics of cancer patients are summarized in Table 1. While the cancer patients and control groups were well matched in age, BMI, heart rate, and blood pressure, there were marked differences in serum TSH (*P* = 0.001) and free T4 (*P* = 0.002). However, there were no differences between groups in the mean quantities of serum free T3 and plasma NT-pro-BNP (Table 2). There were no significant differences in 2D echocardiographic parameters compared with the control group. The LV chamber size and LV systolic function (LVEF and GLS) had no differences. The LV mass index was higher than in the control group, but not significantly (87.6 g/m^2^ versus 79.3 g/m^2^, *P* = 0.134). There were no significant differences in diastolic echocardiographic parameters, such as *E*, *A*, *e*′, *a*′, IVRT, and *E*/*e*′. The GLS of the left ventricle was also not different between the groups (Table 3).

## 4. Discussion

The principal finding of the present study was that TSH oversuppression therapy may be safe in asymptomatic athyreotic young women with DTC (intermediate or low risk of recurrence) during less-than-10-year TSH-suppressive therapy.

### 4.1. Cardiac Dysfunction in Thyrotoxicosis

Thyrotoxicosis can influence cardiac function and structure and clinical symptoms [18]. Tetraiodothyronine (T4) is biologically inactive in target tissues until converted to triiodothyronine (T3). About 80% of circulating T3 comes from the deiodination of T4 in peripheral tissues [19]. Thyroid hormone, especially T3, causes the gene expression of cardiac contractile proteins and channels or transporters related to ion flux, including calcium, by binding to nuclear T3 receptors or directly affects ion transport in a receptor-independent manner in cardiac myocytes [20]. Changes in T3 status mainly influence cardiac action involving cardiac contractility, electrophysiological function, and cardiac structure. Hyperthyroidism, such as Graves's disease and nodular thyroid disease, is characterized biochemically by a low TSH level and elevated T4, T3, or both. Therefore, hyperthyroidism increases cardiac contractility, resting heart rate, tachyarrhythmias, and LV muscle mass and eventually has consequences ranging from diastolic dysfunction to heart failure and atrial fibrillation [18, 21–23]. However, our result shows that cardiac function and structure displayed no significant differences, in women with DTC and thyrotoxicosis caused by thyroxine, compared with a normal control group. When receiving long-term TSH suppression therapy with LT4 based on the TNM stage and risk of recurrence of DTC, patients frequently encounter exogenous subclinical hyperthyroidism or a variable degree of thyrotoxicosis. Traditionally, T3 levels in exogenous thyrotoxicosis were normally maintained by standard LT4 therapy without the inclusion of T3 in patients who had undergone near-total or total thyroidectomy [24]. In this study, the levels of serum FT4 were elevated and levels of TSH were suppressed compared to those in controls. However, there were no differences in the levels of serum FT3 between the cancer and control groups. Peripheral autoregulation of T4-to-T3 conversion appears to be operative at both ends of the T4 spectrum and may serve to maintain or protect serum T3 levels [25]. Endogenous hyperthyroidism increases thyroid hormone production (T4 and T3) and the fraction of T3 relative to T4 in thyroid secretion by the increased activity of type 1 and 2 iodothyronine deiodinase. Although mild thyrotoxicosis had been developed in DTC patients in this study, no significantly different cardiac effects were caused by the normal levels of T3 due to the peripheral autoregulation of T4-to-T3 conversion. Several studies have been published investigating cardiac effects in athyreotic patients with exogenous subclinical hyperthyroidism. Biondi et al. show that long-term levothyroxine therapy was associated with significantly higher LV mass index, increased heart rate, and significantly impaired cardiac reserve and exercise capacity compared to controls. These studies, however, were performed in symptomatic patients (60~100%) and in a heterogeneous group (multinodular goiter and DTC) while all our patients were asymptomatic and formed a homogenous group (women ≤ 45 years with DTC) [8, 9, 26]. Shapiro et al. have shown that patients with minimal symptoms by a score questionnaire had minimal cardiac effects and suggested that if there are no thyrotoxic symptoms during TSH-suppressive therapy, it is not necessary to perform additional cardiac evaluation in DTC patients [11]. Fazio et al. have reported that TSH-suppressive therapy with levothyroxine in DTC patients increases LV mass and causes diastolic dysfunction. However, the DTC patients in that study had a TSH level of less than 0.05 mU/L, while our patients had a serum level of TSH < 0.1 mU/L in the intermediate-risk-of-recurrence group and <0.3 mU/L in the lower-risk-of-recurrence group [27]. Also, the calculation formula of LV mass was different in the above studies (Penn or Devereux), compared with our formula (ASE), which is now widely used. We also analyze the GLS of the LV, as it is known that higher GLS predicts a poor prognosis for decreased LV systolic function, heart failure, and ischemic cardiomyopathy, and we show no significant difference between the TSH suppression group and control subjects [28–30]. This study had several strengths. The athyreotic patients were exclusively DTC patients with low or intermediate risk of recurrence and were all women aged less than 45 years (the most prevalent group in DTC) when they received a total or near-total thyroidectomy. The serum TSH levels were oversuppressed for more than 2 years with LT4 relative to the target levels of TSH that corresponds to their risk of mortality and recurrence based on management guidelines. These enrollment criteria suggest that the oversuppression of TSH frequently encountered during the long-term management of young DTC patients (<45 years old) without heart diseases may be safe for cardiac function and structure, especially for patients with the low or intermediate risk of recurrence. However, this study had some limitations. First, the small number of patients in this study might increase the possibility of beta error (false-negative), which means the failure to detect cardiac effect by TSH oversuppression that is present. A subgroup analysis according to the risk of recurrence and extent of thyroidectomy was not possible. Second, blood samples were collected following the ingestion of LT4 by DTC patients. The serum free T4 levels in DTC patients measured in such a way might be elevated compared to if they were measured prior to ingestion of LT4 [31]. The timing of blood sampling might increase the difference of serum free T4 concentrations between groups. Third, we did not check the thyrotoxic symptom score by standardized questionnaires [32]. Fourth, some of the enrolled DTC patients had comorbid diseases, such as hypertension and anemia, which might affect cardiac structure and function. The control subject was matched to a patient by age, sex, and BMI. We could not match the comorbid diseases exactly between groups. This prevented a direct comparison with other previous studies [8–11]. Fifth, the most current ATA guidelines for diagnosis and management of DTC patients have just been released and direct physicians to adjust TSH suppression based upon ongoing risk stratification and response to therapy making this report less applicable [33].

In conclusion, the present study shows that TSH-suppressive therapy with LT4 in asymptomatic athyreotic women with DTC, especially in the low- or intermediate-risk-of-recurrence categories, may be safe for cardiac function and structure despite intermittent oversuppression during long-term (less than 10 years) TSH-suppressive therapy. Also, this study suggests that if no cardiac symptoms are present in overly suppressed DTC patients, additional cardiac evaluation for cardiac function and structure is not necessary.

## Figures and Tables

**Table 1:** Baseline characteristics of differentiated thyroid cancer patients based on TNM classification and recurrence-risk stratification system.

Age	Thyroidectomy	Cell type	Remnant ablation	TNM system	Recurrence risk	LT4 dose (*μ*g/kg)	TSH suppression (year)	Comorbidity (ongoing medication)
49	Total	Papillary	Yes	T4aN0M0	Intermediate	3.34	6	CML (imatinib)
36	Total	Papillary	No	T2N1aM0	Intermediate	2.20	8	None
54	Near-total	Papillary	Yes	T1bN0M0	Low	2.78	6	PMS (HT)
48	Total	Papillary	No	T2N1aM0	Intermediate	2.55	5	HTN (CCB)
33	Near-total	Hurthle	Yes	T1bN1aM0	Intermediate	2.66	6	None
38	Near-total	Papillary	Yes	T3N1bM0	Intermediate	2.80	6	None
38	Total	Papillary	Yes	T2N0M0	Low	2.79	9	None
50	Near-total	Papillary	Yes	T2N1aM0	Intermediate	3.05	6	None
41	Near-total	Papillary	Yes	T1aN1aM0	Intermediate	2.28	6	None
32	Total	Papillary	Yes	T2N0M0	Low	2.44	7	None
38	Total	Papillary	Yes	T2N1aM0	Intermediate	3.82	9	None
45	Total	Mixed	Yes	T2N0M0	Low	1.67	7	None
51	Near-total	Papillary	Yes	T2N1aM0	Intermediate	2.79	7	IDA (iron)
48	Total	Papillary	Yes	T1bN1aM0	Intermediate	3.62	9	PTB

CCB, calcium channel blocker; CML, chronic myeloid leukemia; HT, hormonal therapy; HTN, hypertension; IDA, iron deficiency anemia; LT4, levothyroxine; PMS, postmenopausal syndrome; PTB, pulmonary tuberculosis; TSH, thyroid-stimulating hormone.

**Table 2:** Clinical and laboratory characteristics of the control group and differentiated thyroid cancer patients receiving long-term (5 to 9 years) levothyroxine suppression therapy.

Parameters	Cancer group (*n* = 14)	Control patients (*n* = 14)	*P* value
Age (years)	42.9 ± 7.2	42.9 ± 6.5	0.637
BMI (kg/m^2^)	23.1 ± 2.1	23.5 ± 1.8	0.198
Heart rate (beats/min)	68.0 ± 9.3	66.1 ± 8.1	0.484
SBP (mmHg)	116.4 ± 10.6	118.6 ± 12.9	0.662
DBP (mmHg)	75.7 ± 6.2	76.4 ± 9.3	0.819
TSH (mU/L)	0.06 ± 0.03	1.58 ± 1.29	0.001
Free T4 (pmol/L)	19.7 ± 5.2	11.5 ± 2.4	0.002
Free T3 (pmol/L)	4.4 ± 0.3	4.3 ± 0.2	0.730
NT-pro-BNP (pg/mL)	59.2 ± 50.3	38.0 ± 19.4	0.198

Data are expressed as mean ± standard deviation. *P* values are calculated by Mann-Whitney test. BMI, body mass index; DBP, diastolic blood pressure; SBP, systolic blood pressure; TSH, thyroid-stimulating hormone.

**Table 3:** Echocardiographic parameters in differentiated thyroid cancer patients and the control group.

Parameters	Cancer group (*n* = 14)	Control group (*n* = 14)	*P* value
LVEDD (mm)	48.6 ± 4.1	48.8 ± 2.5	0.667
LVESD (mm)	30.1 ± 2.4	30 ± 4.1	0.982
LV mass index (g/m^2^)	87.6 ± 20.1	79.3 ± 11.4	0.134
LAVi (mL/m^2^)	26.9 ± 7.2	31.4 ± 7.8	0.178
LVEF (%)	67.8 ± 4.3	68.1 ± 7.6	0.701
*E* velocity (cm/s)	85.1 ± 16.2	79.7 ± 15.5	0.352
*A* velocity (cm/s)	62.8 ± 14.5	68.3 ± 18.2	0.454
*e*′ velocity (cm/s)	9.2 ± 1.6	8.5 ± 1.8	0.239
*a*′ velocity (cm/s)	8.1 ± 1.4	7.9 ± 1.4	0.616
IVRT (millisecond)	94.1 ± 7.7	92.4 ± 4.0	0.832
*E*/*e*′ ratio	9.6 ± 2.6	9.6 ± 2.1	0.756
GLS (%)	−21.6 ± 2.7	−21.2 ± 2.4	0.804

Data are expressed as mean ± standard deviation. *P* values are calculated by Mann-Whitney test. *A*, late diastolic flow of mitral valve; *a*′, late diastolic motion of mitral annulus; *E*, early diastolic flow of mitral valve; *e*′, early diastolic motion of mitral annulus; IVRT, isovolumetric relaxation time; GLS, global longitudinal strain; LAVi, left atrial volume index; LVEDD, left ventricular end-diastolic dimension; LVESD, left ventricular end-systolic dimension; LVEF, left ventricular ejection fraction.
